# Correlation between systemic immune inflammatory index and prognosis of patients with hepatic alveolar hydatid disease and establishment of a nomogram prediction model

**DOI:** 10.3389/fsurg.2022.1019963

**Published:** 2023-01-06

**Authors:** Chen Xiaobin, Yuan Jiaqi, Xu Zhaojun, Pan Mingquan, Zhou Ying, Hou Lizhao, Ren Li, Wang Haijiu, Wang Zhixin, Fan Haining, Chen Lin

**Affiliations:** ^1^ Department of Hepatopancreatobiliary Surgery, Affiliated Hospital of Qinghai University, Qinghai, China; ^2^ Qinghai Research Key Laboratory for Echinococcosis, Qinghai, China; ^3^ Department of Gastrointestinal Surgery, Affiliated Hospital of Qinghai University, Qinghai, China

**Keywords:** systemic immune inflammatory, prognosis, risk factors, nomogram, hepatic alveolar echinococcosis

## Abstract

**Background:**

To explore the evaluation value of systemic immune inflammation index (SII) in the prognosis of patients with alveolar hydatid disease, and establish a nomogram prediction model.

**Methods:**

Collect the clinical data of 351 patients undergoing hepatic alveolar hydatid surgery admitted to the Department of Hepatobiliary and Pancreatic Surgery, Affiliated Hospital of Qinghai University from January 2015 to December 2020, calculate the SII value, and use the receiver operating characteristic curve (ROC curve) to determine According to the optimal clinical cut-off value of SII, patients were divided into two groups with high SII and low SII, and the relationship between SII and clinicopathological factors and prognosis of patients with alveolar echinococcosis was analyzed. Establish a nomogram prediction model based on independent risk factors for patient prognosis, and evaluate the prediction accuracy and discrimination ability of the nomogram through the consistency index (C-index) and calibration curve. The result is through the use of bootstrapping validation with 1,000 re-sampling Method for internal verification.

**Results:**

The ROC curve was used to determine the optimal cut-off value of SII before operation 761.192, and patients were divided into low SII group (*n* = 184) cases and high SII group (*n* = 167) cases. The 1, 3, and 5-year survival rates of patients with hepatic alveolar hydatid in the low SII group and the high SII group were 98.90%, 96.90%, 86.50% and 98.20%, 72.50%, 40.30%, respectively. The survival rate of worm disease patients was significantly better than that of the high SII group, and the overall survival rate difference between the two groups was statistically significant (*P* < 0.001). Multivariate Cox regression model analysis results showed that intraoperative blood loss (HR = 1.810, 95%CI: 1.227–2.668, *P* = 0.003), SII (HR = 5.011, 95%CI: 3.052–8.228, *P* < 0.001), Complications (HR = 1.720, 95%CI: 1.162–2.545, *P* = 0.007) are independent risk factors for the prognosis of patients with alveolar hydatid disease. Draw a nomogram and include statistically significant factors in the multivariate Cox regression model to predict the overall survival rate of patients with alveolar hydatid disease at 1, 3, and 5 years. The survival probability calibration curve is displayed. The nomogram is compared with The actual results have a high degree of agreement. The concordance index (C-index) of the nomogram model in the modeling sample is 0.777, and the C-index in the verification sample is 0.797, indicating that the nomogram model of this study has good accuracy and discrimination.

**Conclusions:**

SII has a clear correlation to the prognosis of patients with alveolar echinococcosis. The nomogram prediction model constructed on this basis is beneficial to the clinically individualized analysis of the patient's prognosis.

## Introduction

Hydatid disease, also known as echinococcosis, is a zoonotic disease prevalent in the world. Currently, the number of infected people is as high as 4 million, and 60 million people are at risk of infection. It has become a serious threat to the public health and health of the world (1). There are two main types of hepatic hydatid disease in humans, cystic echinococcosis (CE) caused by E. alveolar echinococcosis, AE) (2–4). Although the incidence of hepatic alveolar echinococcosis is slightly lower than that of hepatic cystic echinococcosis, it often proliferates by budding or infiltrating, similar to tumor-like growth patterns (e.g., it is easy to invade adjacent organs and pass through blood vessels), lymphatic vessels and biliary tract and other ways of distant metastasis), so the pathogenicity is very strong, the morbidity and mortality rate are high, so it is called “worm cancer”. At present, surgical treatment is the main treatment for hepatic alveolar echinococcosis. However, many postoperative complications often lead to poor prognosis of patients (5, 6). Hepatic alveolar hydatid disease patients with untreated or ineffective treatment have a fatality rate as high as 90% within 10–15 years after diagnosis, which seriously threatens human life and health (7). Therefore, at this stage, relevant researchers continue to explore, aiming to discover new simple, economical and accurate prognostic evaluation indicators similar to PNM classification, so as to better guide clinical treatment.

Studies have shown that the prognosis of tumor patients is related to inflammatory response and immune response, and the relevant circulating immune cells and inflammatory cells involved are mainly neutrophils (N), lymphocytes (L) and platelets (P). The Systemic Immune Inflammatory Index (SII) based on platelets, lymphocytes and neutrophils has been proved to be of great value in evaluating the prognosis of esophageal cancer, breast cancer, liver cancer and pancreatic cancer (8–11). In recent years, relevant studies have shown that inflammation is involved in the pathogenesis of hepatic alveolar echinococcosis. Based on this, this study collected clinical and follow-up data from patients with hepatic alveolar hydatid disease, and analyzed the relationship between SII and hepatic alveolar hydatid disease. To investigate the relationship between clinicopathological characteristics and prognosis of patients with hepatic alveolar echinococcosis, to explore the significance of SII in the prognosis of patients with hepatic alveolar echinococcosis, and to construct a nomogram prediction model for predicting the prognosis of patients with hepatic alveolar echinococcosis to provide clinical decision-making. In accordance with. To our knowledge, this is the first report on the application of nomogram for prognosis prediction in patients with hepatic alveolar echinococcosis.

## Materials and methods

### Data collection

The clinical data of patients with hepatic alveolar hydatid disease treated by hepatobiliary and pancreatic surgery in Qinghai University Affiliated Hospital from January 2015 to December 2020 were collected. Treatment options include radical resection of liver hydatid disease and palliative care. Inclusion criteria: (1) Hepatic alveolar hydatid disease was diagnosed by abdominal B-ultrasound and abdominal CT; (2) Preoperative treatment with albendazole anti-insect drugs was not targeted; (3) Preoperative acute and chronic diseases were not combined. Inflammation, the results of routine blood test were normal; (4) Child-Pugh grading of preoperative liver function was A or B. Exclusion criteria: (1) the postoperative pathological diagnosis was not hepatic alveolar hydatid disease; (2) the medical records were missing or lost to follow-up; (3) complicated with liver cirrhosis and liver tumor; (4) refused surgical treatment. All patients signed informed consent and this study protocol was approved by the Ethics Committee of Qinghai University Affiliated Hospital (batch number: PSL2018006).

### Methods

The results of the first blood collection after admission were collected, and SII was calculated, SII = peripheral platelet × neutrophil/lymphocyte count. According to the optimal cut-off value of SII, patients were divided into two groups: low SII and high SII, and the relationship between SII and clinicopathological factors of patients was analyzed.

### Follow up

All postoperative patients were followed up regularly, and the follow-up period was once every 3–6 months. The contents of follow-up included the current general condition of the patient, the recurrence of hepatic hydatid and the time of recurrence, the postoperative adjuvant therapy and treatment plan, and the time and cause of death of the patient who died. The follow-up deadline was June 1, 2022, when the patient died. Overall survival was defined as the time from postoperative day 1 to death or the end of follow-up.

### Statistical analysis

SPSS 26.0 software and R software were used for statistical analysis of the data, and the *χ*^2^ test was used to compare the qualitative variables between the two groups. The receiver operating characteristic curve (ROC curve) was used to determine the optimal critical value of SII, the Kaplan-Meier method was used to draw the survival curve, and the overall survival time of the two groups of patients was analyzed. Log-rank was used to compare the difference in survival time between the two groups; Cox regression model was used to analyze the relationship between SII and prognosis of patients with hepatic alveolar echinococcosis, and HR and corresponding 95% CI were calculated. According to the results of Cox multivariate analysis, the “rms” package in R software (version 3.6.0) was used to establish a nomogram prediction model (12). Model selection is done through a stepwise selection process following the Akaike Information Criterion (13). In addition, the Harrell Concordance Index (C-index) was used to quantify the discriminative performance of the predicted nomogram (14), and was evaluated by comparing the survival probability between the nomogram prediction and the actual Kaplan-Meier estimate, using a nomogram with 1,000 A sub-resampled bootstrapping validation method is used for internal validation to calculate the relative corrected C-index (15). The larger the C-index, the more accurate the prognosis prediction (16). *P* < 0.05 means the difference is statistically significant.

## Results

### General data

A total of 351 patients met the inclusion criteria, including 211 females (60.01%) and 140 males (39.89%), aged 7–73 years, with an average of (36.98 ± 12.5) years. All 351 patients were diagnosed with hepatic alveolar hydatid disease, and all patients were discharged after treatment. Among all patients, 296 patients (84.3%) received radical resection of liver hydatid, and 55 patients (15.7%) received palliative treatment. As of June 1, 2022, all 351 patients were followed up with a median follow-up time of 45 months, of which 110 (31.3%) died and 241 (68.7%) were still alive.

### Determination of the optimal critical value of preoperative SII

**Figure 1:**
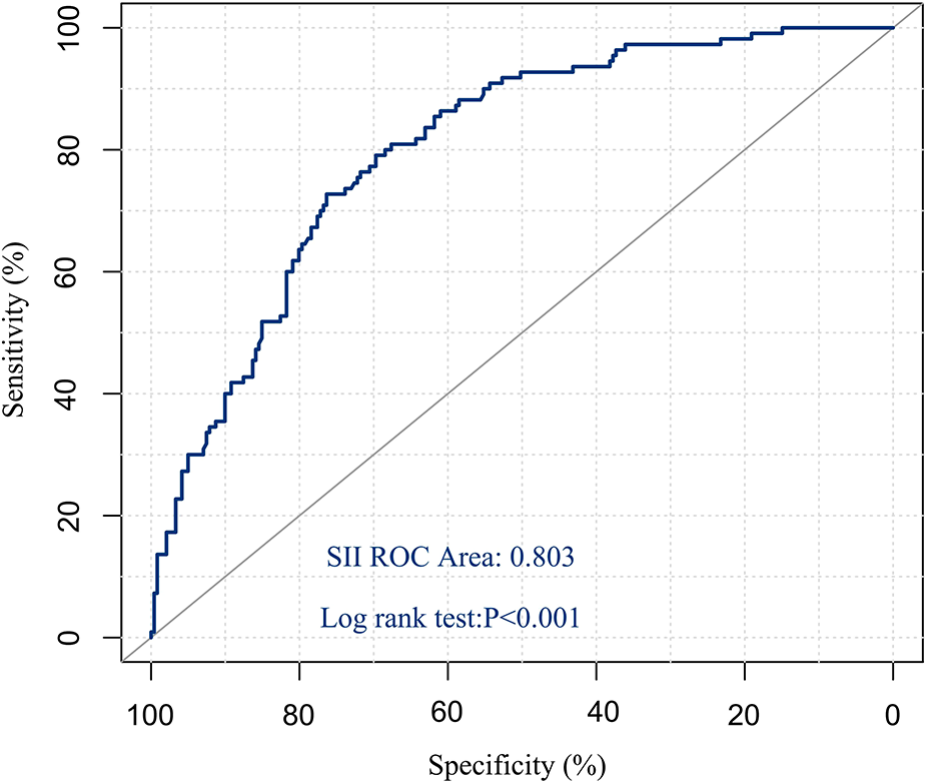
ROC curve of preoperative SII predicting overall survival in patients with hepatic hydatid disease.

Drawing the ROC curve of SII, the area under the ROC curve was 0.803 (95%CI: 0.757–0.850, *P* < 0.001), the Youden index was 0.485, and the corresponding optimal critical value was 761.192, which was sensitive to assessing the survival of postoperative patients. The specificity was 80.9% and the specificity was 67.6% (Figure 1). According to this critical value, 351 patients were divided into two groups, including 184 patients in the low SII group (SII ≤ 761.192) and 167 patients in the high SII group (SII > 761.192).

### Relationship between SII and clinicopathological factors in patients with hepatic alveolar echinococcosis

SII was related to surgical method, intraoperative blood loss, hydatid stage, Child-Pugh grade, complications, ALT, AST, TBil, ALB, ALP, PT, platelet, white blood cell count and metastasis (all *P* < 0.05). However, it was not related to age, gender and neutrophils (all *P* > 0.05) (Table 1).

**Table 1:** Relationship between SII and clinicopathological factors in patients with hepatic alveolar echinococcosis.

Variables	Low SII	High SII	*χ* ^2^	*P*
	(*n *= 184)	(*n *= 167)		
Age (year)	59		0.03	0.862
≤30		55		
>30	125	112		
Sex			0.018	0.894
Male	74	66		
Female	110	101		
Surgical approach			34.514	<0.001
Radical treatment	176	120		
Palliative care	8	47		
Intraoperative blood loss (ml)			50.751	<0.001
<1000	155	81		
≥1000	29	86		
PNM stages			28.971	<0.001
I, II	103	46		
≥III	81	121		
Child-Pugh			13.186	<0.001
A	113	81		
B	71	86		
Complication			7.516	0.003
Yes	116	81		
None	68	86		
ALT (U/l)			16.272	<0.001
≤40	122	75		
>40	62	92		
AST (U/l)			17.918	<0.001
≤40	129	80		
>40	55	87		
TBil (umol/l)			27.986	<0.001
≤34.2	146	88		
>34.2	38	79		
ALB (g/l)			30.931	<0.001
≤35	71	114		
>35	113	53		
ALP (U/l)			42.029	0.02
≤150	94	30		
>150	90	137		
PT (s)			6.947	0.001
≤16	166	123		
>16	18	44		
NE (×10^9^/l)			16.517	0.232
≤6.3	171	138		
>6.3	12	29		
PLT (×10^9^/l)			5.054	0.025
≤300	125	78		
>300	59	89		
WBC (×10^9^/l)			11.445	0.001
≤10	170	138		
>10	8	25		
Whether to transfer			28.971	<0.001
Yes	103	46		
None	81	121		

### Relationship between SII and overall survival in patients with hepatic alveolar echinococcosis

**Figure 2:**
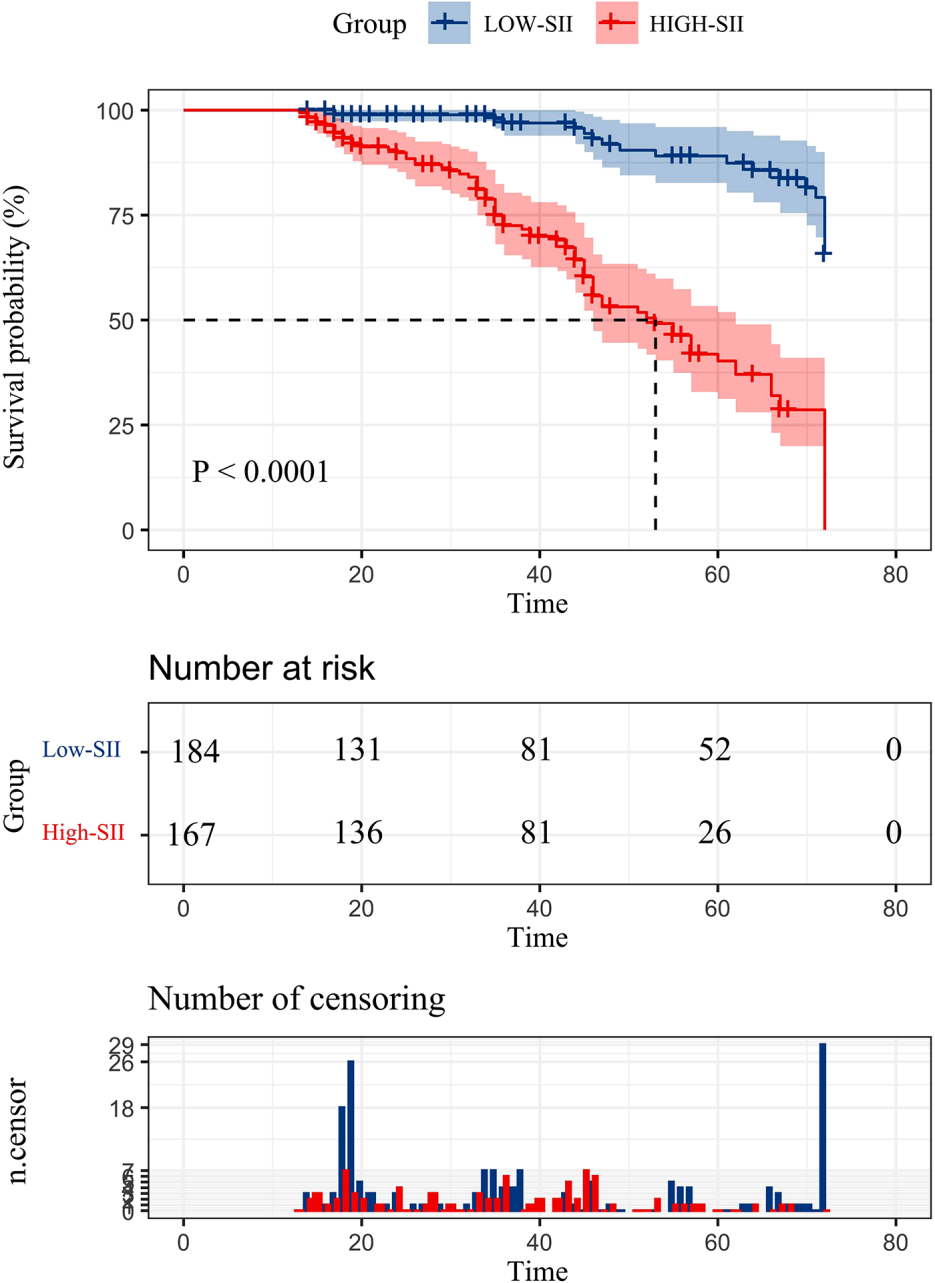
Postoperative overall survival curve of patients with hepatic alveolar hydatid disease in two groups.

The cumulative survival rate in the low SII group was >50%, and the average survival time was 68.256 months (95%CI: 66.255–70.257); the cumulative survival rate in the high SII group was <50%, and the average survival time was 51.28 months (95%CI: 47.990–54.570), and the median survival time was 53 months (95%CI: 45.324–60.676). The 1-, 3-, and 5-year survival rates in the low SII group were 98.90%, 96.90%, and 86.50%, respectively; the 1-, 3-, and 5-year survival rates in the high-SII group were 98.20%, 72.50%, and 40.30%, respectively, and the survival rates in the low SII group were It was better than the high SII group, and there was a statistically significant difference in the overall survival rate between the two groups (*P* < 0.001) (Figure 2).

### Analysis of influencing factors for prognosis of patients with hepatic alveolar hydatid disease

Univariate analysis showed that surgical methods, intraoperative blood loss, ALB, SII, and complications were all prognostic factors for patients with hepatic alveolar hydatid disease (all *P* < 0.05). Moderate bleeding volume (HR = 1.810, 95%CI: 1.227–2.668, *P* = 0.003), SII (HR = 5.011, 95%CI: 3.052–8.228, *P* < 0.001), complications (HR = 1.720, 95%CI: 1.162–2.545, *P* = 0.007) was an independent risk factor for the prognosis of patients with hepatic alveolar hydatid disease, as shown in Table 2.

**Table 2:** Univariate and multivariate analysis on the survival of patients with hepatic alveolar hydatid disease.

Variables	Univariate analysis		Multivariate analysis	
	HR (95%CI)	*P*	HR (95%CI)	*P*
Age (≤30 vs. >30)	1.164 (0.765–1.772)	0.479		
Sex (Male vs. Female)	0.875 (0.599–1.278)	0.490		
Surgical approach (radical treatment vs. palliative care)	1.980 (1.294–3.032)	0.002		
Intraoperative blood loss (≤1,000 vs. >1,000)	2.705 (1.856–3.942)	0.001	1.810 (1.227–2.668)	0.003
PNM stages (I, II vs. ≥III)	1.100 (0.692–1.747)	0.687		
Child-Pugh (A vs. B)	2.013 (1.214–4.571)	0.053		
ALT (≤40 vs. >40)	0.956 (0.656–1.392)	0.813		
AST (≤40 vs. >40)	1.116 (0.767–1.623)	0.566		
ALB (≤35 vs. >35)	0.654 (0.436–0.981)	0.040		
TB (≤34.2 vs. >34.2)	1.079 (0.737–1.579)	0.696		
PT (≤16 vs. >16)	0.928 (0.577–1.494)	0.759		
SII (≤761.192 vs. >761.192)	6.361 (3.935–10.281)	0.001	5.011 (3.052–8.228)	0.001
Whether to transfer (none vs. yes)	1.100 (0.692–1.747)	0.687		
Complication (none vs. yes)	2.410 (1.641–3.539)	0.001	1.720 (1.162–2.545)	0.007

### Predictive effect of nomogram prediction model on cumulative survival rate of patients with hepatic alveolar hydatid disease

**Figure 3:**
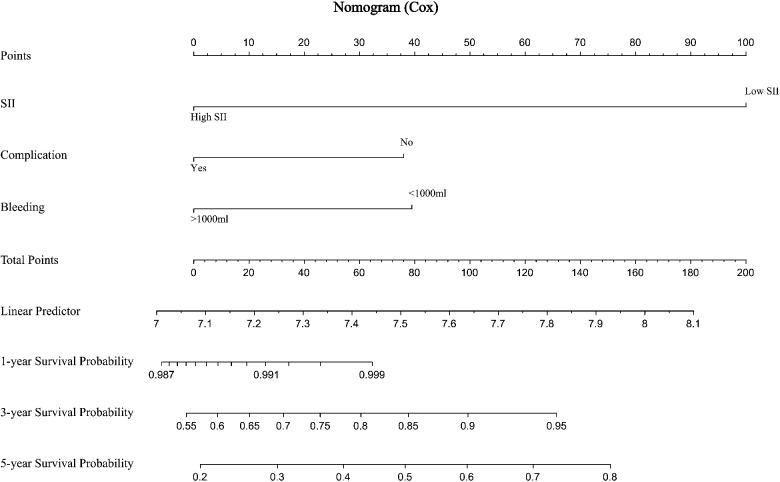
Prediction of 1-, 3-, and 5-year survival rates of patients with hepatic alveolar hydatid disease by nomogram.

**Figure 4:**
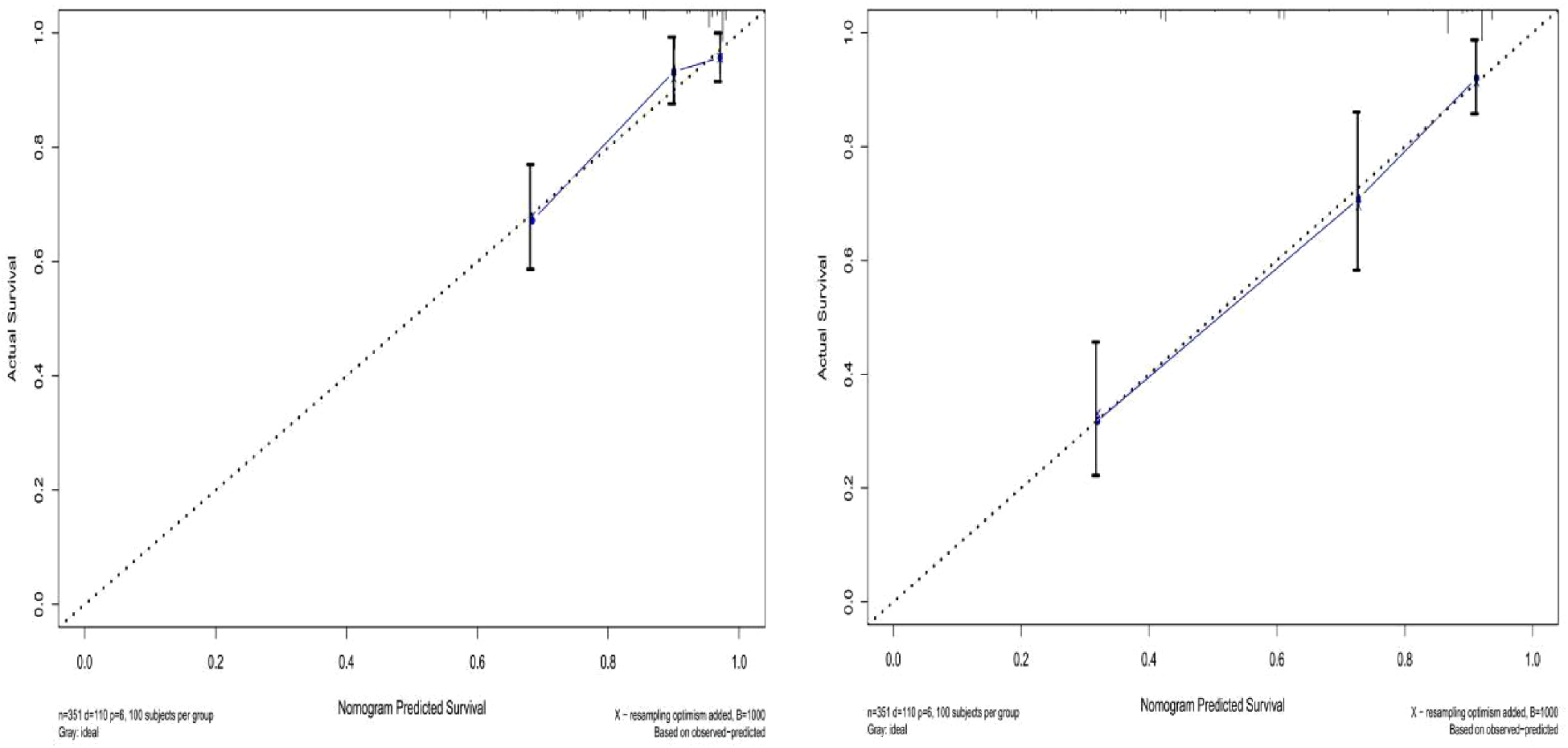
Survival calibration curve of patients with hepatic alveolar echinococcosis. A is 3-year survival, B is 5-year survival, and the probability of nomogram-predicted overall survival is plotted on the *x*-axis; actual overall survival is plotted on the *y*-axis.

**Figure 5:**
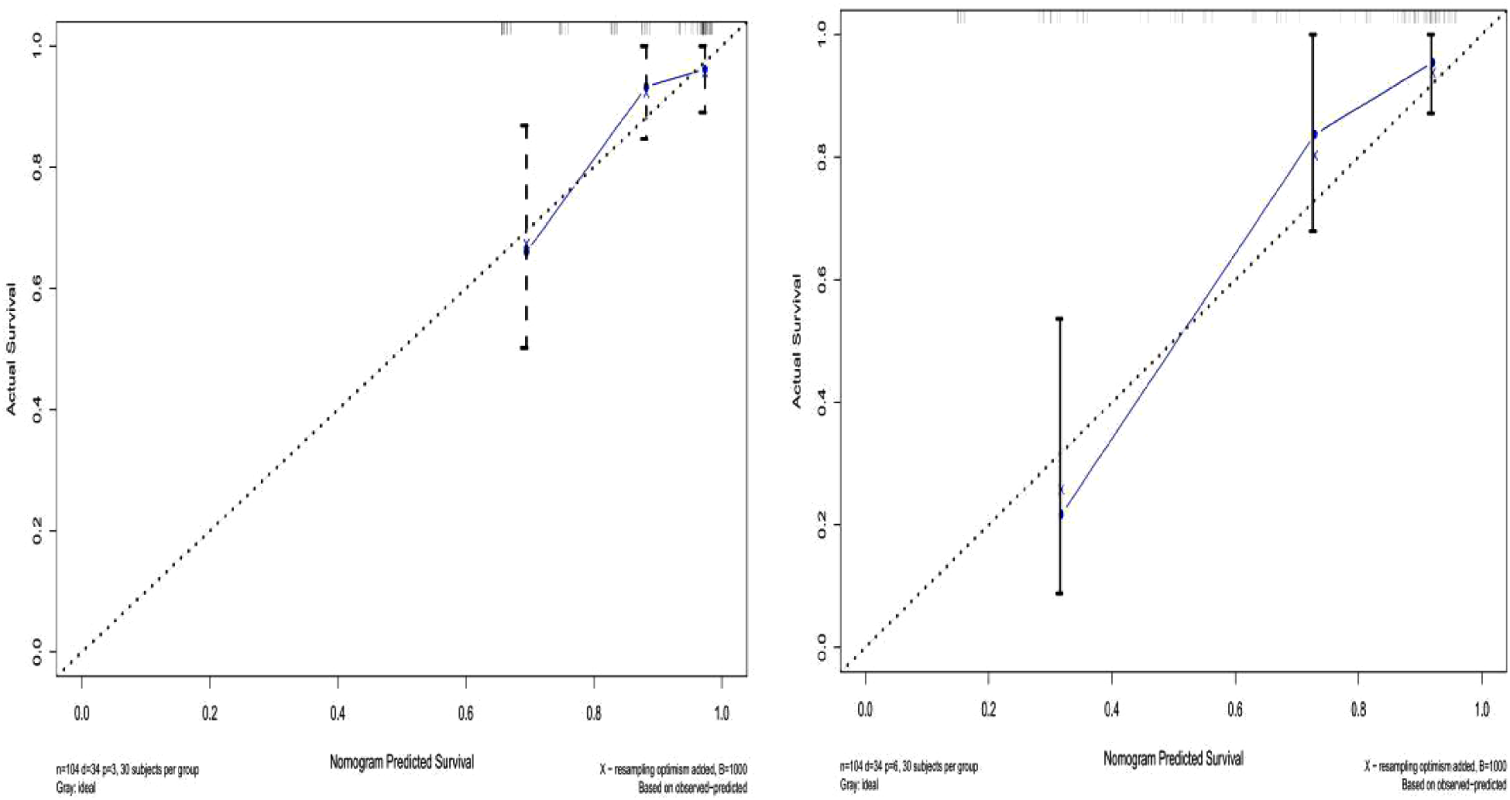
Validation set survival calibration curve for patients with hepatic alveolar echinococcosis.

The prediction effect of the cumulative survival rate of patients with hepatic alveolar hydatid disease was displayed by drawing a nomogram, and the prediction model was established according to the independent risk factors (intraoperative blood loss, SII, complications) screened out by the Cox regression model, and listed in the column The line plot shows that the total score for each patient was assigned by drawing a vertical line from the corresponding point of each predictor variable to the score table, and adding these scores to obtain the total score, for patients with alveolar hydatid disease The 1-year, 3-year, and 5-year survival rates were predicted, and the consistency index (C-index) was 0.777 (Figure 3). The calibration curves are all very close to the ideal curve (Figure 4), indicating that the predicted values obtained from the nomogram prediction model can better represent the actual values. In the internal validation cohort, the validation C-index was 0.797 by bootstrapping validation analysis, which indicates that the model has good discriminative power. The calibration curve also performed well in the validation set, as shown in Figure 5.

## Discussion

In recent years, more and more studies have shown that inflammatory response plays an important role in disease progression and is also involved in the regulation of host immune function. Some biomarkers such as C-reactive protein, α1-acid glycoprotein, neutrophils Neutrophil-lymphocyte ratio (NLR) and platelet-lymphocyte ratio (PLR) are closely related to the prognosis of cancer patients and have potential clinical significance for monitoring the prognosis of cancer patients (17, 18). Among them, SII is a comprehensive index based on lymphocytes, platelets and neutrophils, reflecting the body's inflammatory response index. Compared with other indexes, this inflammatory index reflects the balance between the host's immune state and inflammatory state more comprehensively. It has been proved that It is a valuable prognostic indicator for tumor patients (19, 20). Recent studies (11, 17, 18) have shown that high levels of SII are associated with poor prognosis in malignancies such as hepatocellular, gastric, breast and pancreatic cancers.

In this study, univariate and multivariate Cox regression analysis confirmed that intraoperative blood loss, complications, and SII were independent risk factors affecting the prognosis of patients with alveolar hydatid disease, which had important clinical significance. Agid et al. (21) found that the risk of complications was significantly related to the prognosis of hepatic alveolar echinococcosis, reflecting the survival rate of patients to some extent, which was consistent with the results of this paper, by discussing the relationship between postoperative complications of hepatic alveolar echinococcosis, surgical methods and Child-Pugh grading of liver function before operation. In addition, the results of this study suggest that the amount of intraoperative blood loss is the key factor affecting the prognosis of patients with hepatic alveolar echinococcosis, which is consistent with the results of Ma Hailin (22). By analyzing the influencing factors of postoperative survival time of patients with end-stage hepatic alveolar echinococcosis treated by excised hepatectomy and autotransplantation, Ma Hailin found that the amount of intraoperative blood loss (RR = 0.096, 95% CI: 0.020–0.450, *P* = 0.003). In addition, the liver is an important blood storage organ, and excessive intraoperative blood loss will lead to postoperative liver function decline to a certain extent. Hanazaki et al. (23) pointed out that for radical surgery of hepatocellular carcinoma (HCC), even if a small amount of blood is infused during operation, the risk of postoperative recurrence of HCC may increase. Subsequently, Eiji Tsujita et al. (24) found that intraoperative blood loss was an independent risk factor for the prognosis of recurrent hepatocellular carcinoma (HCC) by studying the adverse prognostic factors of the second operation.

This study shows that the survival time of patients with hepatic alveolar echinococcosis in the high SII group is significantly shorter than that in the low SII group. The author considers that the higher level of SII mostly represents the higher level of neutrophils and platelets and the lower level of lymphocytes. On the one hand, platelet-derived TGFβ1 can induce epithelial mesenchymal transition and synergistically promote the transfer of worms by activating NF-κB and TGFβ/Smad pathways (25); on the other hand, lymphocytes, as immune cells, can inhibit the growth and metastasis of worms (26); finally, neutrophils can aggravate local inflammatory reaction by activating complement and coagulation system, leading to tissue damage. Parasites can induce neutrophils to have oxidative and non-oxidative killing reactions, which will aggravate the progress of the disease (27). These are the main factors leading to poor prognosis of patients with hepatic alveolar echinococcosis.

In this study, a nomogram was constructed according to the independent risk factors of prognosis screened by Cox regression model, and the prognosis of patients with hepatic alveolar echinococcosis was predicted and evaluated by nomogram. The accuracy of nomogram is evaluated by C-index and calibration chart, which proves that nomogram has high predictive value. The nomogram provides the specific scores of each influencing factor, and the final probability of the outcome can be predicted by adding the scores of each factor. At the same time, the nomogram transforms the complex regression equation into a simple visual graph, which makes the prediction results of the prediction model more readable. Medical staff can predict the prognosis of patients with hepatic alveolar echinococcosis more quickly and conveniently according to nomograms, which is conducive to prevention work in advance and has high clinical application value.

There are some limitations in this study. On the one hand, the sample size of this study is small, and the regression model constructed may have some bias, and there is no test set for external verification of survival model and nomogram. Therefore, further samples should be collected for further research in the future. In addition, it is not clear whether the high level of SII before operation means promoting or inhibiting peripheral neutrophils, lymphocytes and platelets, and the related mechanism needs further research to confirm.

In conclusion, the results of this study indicate that SII is expected to be a prognostic indicator for patients with hepatic alveolar hydatid disease due to its simplicity, non-invasiveness, and low cost. In addition, the nomogram has high clinical application value, which can intuitively predict the prognosis of patients with hepatic alveolar hydatid disease, and help clinicians formulate or adjust a reasonable diagnosis and treatment plan in a timely manner.

## Data Availability

The raw data supporting the conclusions of this article will be made available by the authors, without undue reservation.
